# Validity and reliability of an electromyography-based similarity index to quantify lower extremity selective voluntary motor control in children with cerebral palsy

**DOI:** 10.1016/j.cnp.2022.03.003

**Published:** 2022-03-17

**Authors:** Julia Balzer, Annina Fahr, Jeffrey W. Keller, Marietta L. van der Linden, Thomas H. Mercer, Hubertus J.A. van Hedel

**Affiliations:** a Swiss Children’s Rehab, University Children's Hospital Zurich, Zurich, Switzerland; b Centre for Health, Activity and Rehabilitation Research, Queen Margaret University, Edinburgh, United Kingdom; c Children’s Research Center, University Children’s Hospital Zurich, University of Zurich, Zurich, Switzerland

**Keywords:** Motor control, Cerebral palsy, Clinimetric properties, Surface EMG, Outcome measure

## Abstract

**Objective:**

To quantify selective voluntary motor control (SVMC) objectively and more precisely, we combined the “Selective Control Assessment of the Lower Extremity” (SCALE) with surface electromyography. The resulting Similarity Index (SI) measures the similarity of muscle activation patterns. This study evaluated the preliminary validity and reliability of this novel SI_SCALE_ measure in children with cerebral palsy (CP).

**Methods:**

We investigated concurrent validity by correlating the SI_SCALE_ of 24 children with CP (median age 10.6 years) with comparator assessments. For discriminative validity, the patients’ SI_SCALE_ scores were compared to 31 neurologically intact age-matched peers. Test-retest reliability was quantified using intraclass correlation coefficients (ICC) and minimal detectable change (MDC) values.

**Results:**

The SI_SCALE_ correlated strongly with the SCALE (ρ = 0.90, p < .001) and the Gross Motor Function Classification System (ρ = −0.74, p < .001). SI_SCALE_ scores were significantly lower in children with CP compared to healthy peers. Test-retest reliability appeared good (for the more and less affected leg, ICC ≥ 0.84, and MDC ≤ 0.17).

**Conclusions:**

Validity and reliability of the SI_SCALE_ leg and total scores lay within clinically acceptable ranges. Further clinimetric analyses should include responsiveness.

**Significance:**

A neurophysiology-based assessment could contribute to a more refined assessment of SVMC impairments.

## Highlights

• The SI_SCALE_ is a new electromyography-based measure to quantify selective voluntary motor control.
• There is a need for precise, interval-scaled measures of selective voluntary motor control in children with cerebral palsy.
• Concurrent and discriminative validity of the new measure was affirmed and test–retest reliability was acceptable.

## 1 Introduction

A loss of selective voluntary motor control (SVMC) is a common negative motor sign in patients with upper motor neuron (UMN) lesions (Sanger et al., 2006, Cahill-Rowley and Rose, 2014). SVMC is defined as the ability “to isolate the activation of muscles in a selected pattern in response to demands of a voluntary movement or posture’’ (Sanger et al., 2006). Pathophysiologically, impaired SVMC is primarily related to injuries of the corticospinal tract, which are commonly found in children with cerebral palsy (CP). Symptomatically, the UMN damage alters the motor units’ firing properties and impacts voluntary muscle activation and overall motor control.

The International Classification of Functioning, Disability and Health-Children & Youth version lists a loss of SVMC as a core impairment for children with CP as it can negatively influence other body functions and activities (Schiariti and Mâsse, 2015). Indeed, several studies have shown that in addition to other common impairments of children with CP (e.g., spasticity, contractures, muscle weakness), impaired selective control of the lower extremities has a strong and negative influence on gross motor function (Vos et al., 2016, Balzer et al., 2018). Training of SVMC, based on the theory of motor learning and neuroplasticity, is known to occur in neurologically intact people (e.g., athletes or musicians). However, the trainability of SVMC in patients with UMN lesions is still controversial (Levin et al., 2009, Nudo, 2013). In addition, intervention studies targeting SVMC are rare and heterogeneous in terms of the patient population, the intervention, and outcome measures (Everaert et al., 2010, McNeal et al., 2010, Fahr et al., 2020).

Adequate outcome measures are required to increase our understanding of impairments in SVMC, the impact of SVMC on overall motor functioning, and its trainability. However, there is a lack of evidence for the clinimetric properties for most of the measures currently used to assess SVMC of the lower extremities. In a review from 2017, four clinical assessments and three laboratory-based measures of lower limb SVMC were identified (Balzer et al., 2017). Overall, the Selective Control Assessment of the Lower Extremity (SCALE) (Fowler et al., 2009) had the highest level of evidence for its validity and reliability. The SCALE has an ordinal scale and rates selective movement around a single joint as normal (2 points), impaired (1 point), or unable (0 points). It was initially developed as a diagnostic tool (Fowler et al., 2009). Therefore, the SCALE might lack resolution for a fine-graded assessment of SVMC and have limited responsiveness for longitudinal intervention studies (Dobson, 2010).

To advance the measurement of SVMC, previous studies have suggested combining the SCALE testing procedure with another more precise, objective, and interval-scaled measure (Dobson, 2010, Zwaan et al., 2012, Balzer et al., 2016). Surface electromyography (sEMG) has been considered a promising candidate, as it allows investigating motor control at the muscular level and can be recorded while performing the SCALE.

Lee et al. (2004) proposed an algorithm for a quantitative analysis of sEMG patterns to extract details about the relative distribution of muscle activity. The so-called similarity index (SI) measures how much a patient’s multichannel sEMG pattern during a defined voluntary movement resembles the pattern of a reference group. In our case, the SI could assess SVMC by quantifying how similar the muscle activation pattern of patients during the SCALE is to the pattern of a healthy adult reference group.

The aim of this study was to investigate the preliminary validity and reliability of the SI_SCALE,_ i.e., the SI calculated from sEMG that was recorded when participants performed the SCALE. We conducted this clinimetric study in line with recommendations from the COSMIN (COnsensus-based Standards for the selection of health Measurement Instruments) initiative (Mokkink et al., 2019). To establish concurrent validity of the SI_SCALE_, we formulated *a priori* several hypotheses: We expected that the SI_SCALE_ shows a strong positive correlation (Spearman’s rank correlation ρ ≥ 0.70) with the SCALE, and a lower correlation with gross motor function. In line with previous studies (Fowler et al., 2010, Balzer et al., 2016), we also investigated discriminative validity and hypothesized that the SI_SCALE_ would significantly differ between i) children with CP and neurologically intact peers; and ii) the less and more affected limb. Finally, test–retest reliability should be at least moderate, with Intra-class Correlation Coefficients (ICC) exceeding 0.65 (similar to Lim and Sherwood, 2005), and acceptable absolute measurement errors, i.e., minimal detectable changes.

## 2 Methods

### 2.1 Participants

First, in- and out-patients of the Swiss Children’s Rehab Center were recruited using convenience sampling from June 2017 until March 2018. According to recommendations for the sample size for the assessment of validity, we aimed to include 30 children with CP (Mokkink et al., 2019). Inclusion criteria were: a diagnosis of spastic or mixed CP, aged between five and 20 years, and the ability to follow simple instructions. Children with a primarily dyskinetic or ataxic impairment, high variability in muscle tone or spasticity due to medication cessation or epilepsy, or who had a botulinum toxin injection to muscles of the lower extremities within the last six months or any surgical correction of the lower limbs within the previous year were excluded.

Second, to establish discriminative validity, neurologically intact children were recruited. We included children and youths aged six to 18 years without any medical history of neurological or orthopedic diagnosis of the lower extremity.

Third, as the calculation of the SI requires reference muscle activation patterns to which the tested person is compared, we recruited neurologically intact adults by convenience sampling. Adults between 18 and 50 years without central or peripheral neurological injury provided these patterns reflecting mature (fully developed) SVMC. We selected adults as a reference group because young neurologically intact children may also show indicators of reduced SVMC, e.g., mirror movements (Koerte et al., 2010, Riddell et al., 2019). The ethical committee of the Canton of Zurich approved the study (KEK-ZH-Nr.2011-0404/PB_2016-01843). Parents and participants provided informed consent.

### 2.2 Measurement procedures and measures

Three experienced testers (one neuro-pediatric physiotherapist and two human movement scientists) carried out the tests. Each measurement session followed a standardized procedure and lasted less than one hour.

Gross motor control of children with CP was categorized according to the Gross Motor Function Classification System (GMFCS), ranging from level “I” to “V” – higher to lower motor abilities. To gain information about the degree of spasticity in the flexor and extensor muscles around the hip, knee, and ankle joint, the Modified Ashworth Scale (MAS) was applied. Scoring 0 means no velocity-dependent increase in muscle tone in response to passive movement, while a score of 4 indicates rigid in flexion or extension (Bohannon and Smith, 1987). In addition, we determined the dominant or less affected leg by asking participants which foot they use to kick a ball (Chapman et al., 1987). For children with CP, we also relied on the diagnosis (e.g., in cases of unilateral spastic CP).

**Fig. 1:**
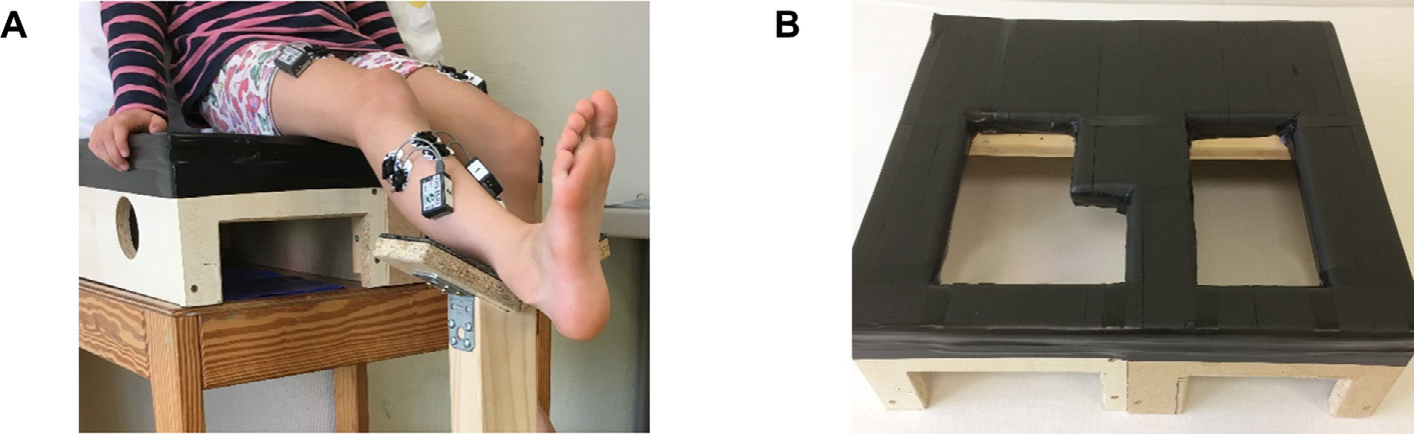
SI_SCALE_ testing setup. A: Picture of a participant equipped with 10 sEMG sensors to record muscle activity patterns during the SCALE. For the foot movements, a standardized lower leg support was used for the tested leg. B: Wooden seat with special openings for the sEMG sensors of the m. semitendinosus.

*SCALE and SI_SCALE_ procedures:* Self-adhesive Ag/AgCl dual snap gel electrodes (Noraxon Inc., Scottsdale, Arizona, United States) with a diameter of 10 mm and an inter-electrode distance of 20 mm were applied bilaterally following the SENIAM (Hermens et al., 1999) guidelines to record sEMG of the following muscles: m. tibialis anterior, m. peroneus longus, m. rectus femoris, m. gastrocnemius medialis, and m. semitendinosus. We selected those muscles that were primarily responsible for the movements tested in the SCALE. The participant was then positioned on a custom-made wooden seat, which had been furnished with special openings for the sEMG sensors of the m. semitendinosus (Fig. 1A and B). EMG data acquisition was performed with a 16-channel Myosystem 1400A (Noraxon Inc., Scottsdale, Arizona, United States), with a sampling frequency of 1500 Hz and a bandwidth of 30–500 Hz.

The SCALE assesses the ability to selectively control reciprocal movements of the hip, knee, ankle, subtalar (STJ), and toe joints (Fowler et al., 2009). The child was asked to perform specific and bidirectional (e.g., knee extension and flexion) movements at each joint. The desired speed is one second per movement direction (e.g., 2 s for extending and flexing a joint), guided via a verbal count of the tester. Each joint movement was repeated three times in one smooth action. The participants were instructed to focus on the target joint movement and relax all other muscles as much as possible. The movements were video recorded and later scored by always the same physiotherapist according to the manual.

We performed the SI_SCALE_ with minor adjustments compared to the SCALE manual (Fowler et al., 2009). (i) We tested all movements in sitting position and in the order knee, ankle, subtalar, and hip joint to minimize positional changes, which would interfere with sEMG measurements. (ii) To standardize the support of the lower leg during the ankle and STJ movements, the lower leg was positioned on a pedestal just proximal to the ankle joint (Fig. 1A). Pilot testing had revealed high variability of (isometric) muscle activity in the m. rectus femoris if the calf was supported manually by the examiner (as described in the SCALE manual). This would have affected the index but would not necessarily reflect SVMC of the participant.

For investigating test–retest reliability of the SI_SCALE_ in children with CP, the measurement was repeated under similar conditions (same testers, time of day, room). To have stable yet independent measurements, assessments were repeated 7–15 days later, and data were recorded on a new, blank collection form.

### 2.3 Data processing

All sEMG signals were filtered using a 20 Hz high pass filter (finite impulse response filter). An infinite impulse response 50 Hz rejection filter was only used for channels that showed 50 Hz noise due to electronic devices. The position of and reason for movement artifacts detected during the visual inspection were documented for further data processing. Artifacts occurred either due to sEMG-cable contact with the seat or participants touching the sEMG sensor with their hands.

Further sEMG data analysis was performed using Matlab (version 2017b). First, movement artifacts were corrected by removing these segments (minimal cutting) from the data. Second, we corrected for background sEMG activity. Initially, we had recorded a 1 min rest baseline period prior to the measurement. The children were asked to keep their muscles relaxed during that period. However, we noted that the muscle activity levels during this baseline trial were in many participants higher than during some instances of the SI_SCALE_ measurements. Therefore, we searched for the lowest activity values observed over three seconds for each of the 10 muscles recorded at any time during the whole measurement and used these to correct the sEMG data.

**Fig. 2:**
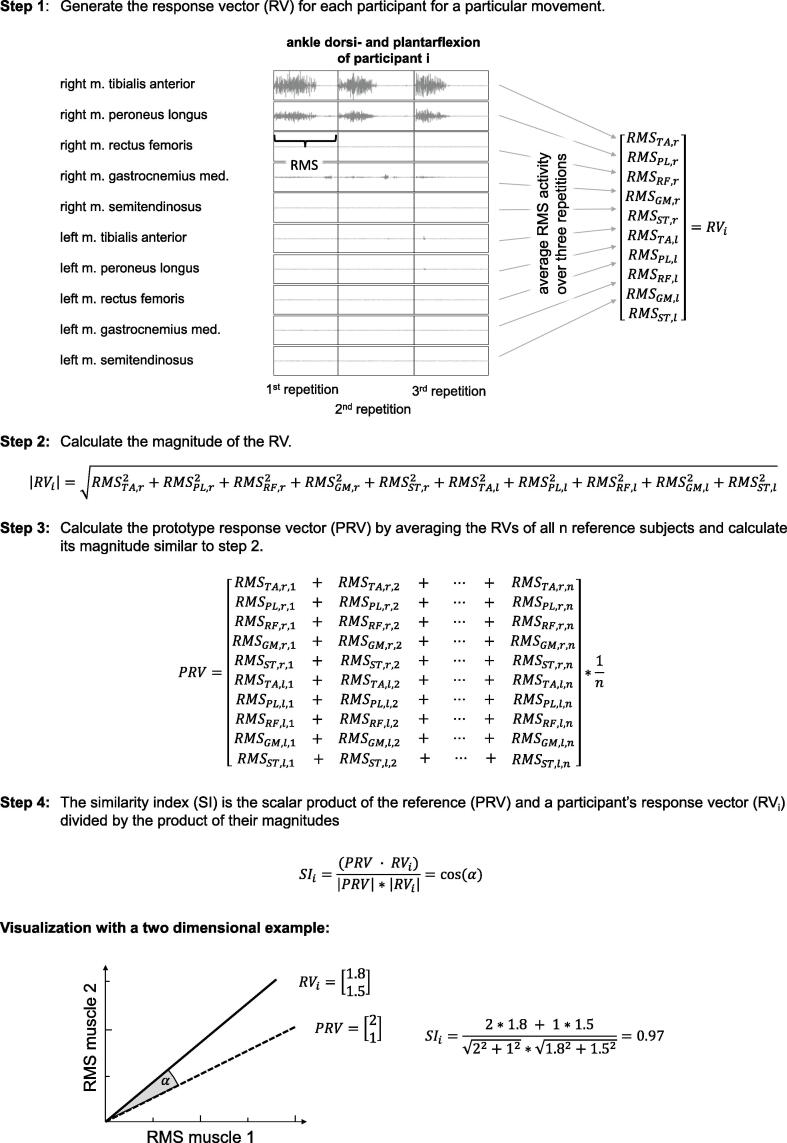
Calculation of the similarity index. Step 1: The response vector (RV) consists of the RMS activity of each of the 10 muscles, averaged over the three movement repetitions, and was derived for each of the four joint movements per leg. The start and end of each movement repetition was identified in the video. Step 2: The magnitude of the RV is calculated by taking the square root of the sum of squares of the RMS of the 10 muscles, and represents the length of this vector. Step 3: The prototype response vector (PRV) is computed by averaging the response vectors of the n = 31 neurologically intact adults elementwise. Step 4: The SI is the scalar product of the reference (PRV) and a participant’s response vector (RV_i_) divided by the product of their magnitudes. It ranges from 0 to 1. Values close to 1 indicate that the participant’s SVMC is very similar to that of the reference. Abbreviations: RV_i_: response vector of participant i, PRV: prototype response vector, SI: similarity index, RMS: root mean square, TA: m. tibialis anterior, PL: m. peroneus longus, RF: m. rectus femoris, GM: m. gastrocnemius medialis, ST: m. semitendinosus; r: right; l: left.

Next, we calculated the root mean square value (RMS) of the sEMG over each movement repetition (identified by event markers) and averaged them across the three repetitions for each joint movement (Step 1 in Fig. 2). Placement of event markers identifying the start and end of each of the three movement repetitions relied on the simultaneously recorded video. Therefore, the length of the analyzed time window was 6 s (3 repetitions of 2 s for extension and flexion) but varied according to the actual movement speed of the participant. Then, the SI was calculated using the equations described by Aslan et al. (2013) and illustrated in steps 2 to 4 of Fig. 2.

In summary, the SI quantifies the similarity between the sEMG activation pattern of the participant (patient or healthy child) and the reference derived from the neurologically intact adult group. For this vector analysis method, the mean RMS of all 10 muscles recorded during the voluntary motor task (SCALE ankle dorsi- and plantarflexion in the example) were combined to a 10-dimensional vector whose direction was compared to the reference vector via the scalar product. Thereby, an SI value of 1 represents ‘normal’ SVMC (i.e., completely similar to the reference), whereas values close to 0 indicate ‘impaired’ SVMC.

### 2.4 Statistical analysis

We applied non-parametric tests to evaluate our *a priori* formulated research hypotheses because the Shapiro-Wilk tests showed that the data were not normally distributed. Results are presented for each individual joint, the mean SI of the less and more affected leg, and the overall mean SI.

Concurrent validity was evaluated by calculating Spearman‘s rank correlation coefficients (ρ) between the SI_SCALE_ and the original SCALE for the total scores (summed SCALE joint scores: 0–16) and for each limb (summed joint scores per leg: 0–8), as well as between the overall mean SI_SCALE_ score and the GMFCS level. For the individual joints, we calculated Kendall’s tau-b rank correlation coefficients (τ) between the SI_SCALE_ and the SCALE. It is specifically designed to handle ties in the data, which are frequent due to the few levels of the SCALE joint score (0–2).

We performed several analyses to determine the discriminative validity of the SI_SCALE._ Firstly, to investigate whether the SI_SCALE_ was different between children with CP and their neurologically intact peers, Mann-Whitney U tests resolved differences in SI_SCALE_ scores for separate joints, limb scores, and the total score. Secondly, we applied Wilcoxon signed-rank tests to investigate differences between the less and more affected leg within a group of children with either bilateral or unilateral leg involvement. Lastly, we computed receiver operating characteristics (ROC) to investigate the ability of the SI_SCALE_ to distinguish normal SVMC (SCALE = 2) from impaired SVMC (SCALE 1 or 0). We included the data from all joints, calculated the area under the ROC curve, and determined the optimal cut-off point using the Youden-Index, i.e., we identified the SI_SCALE_ value with the best combined sensitivity and specificity. Alpha was set at 0.05 (two-tailed) and corrected for multiple comparisons by Bonferroni adjustments.

Relative test–retest reliability was evaluated by ICCs based on a two-way random effects model based on absolute agreement (ICC (2,1) according to Shrout and Fleiss nomenclature) (Koo and Li, 2016) and corresponding 95% confidence intervals. Absolute reliability was determined by the standard error of measurement (SEM = √(σ_t_^2^ + σ_e_^2^), σ_t_ = variance of trial, σ_e_ = variance of residual error) and the minimal detectable change (MDC = SEM × √2 × 1.96) (de Vet et al., 2006, Haley and Fragala-Pinkham, 2006). We tested for systematic errors between test and retest scores with Wilcoxon signed-rank tests.

All statistical analyses were performed with R statistical package version 3.5.1 (R Core Team, 2018) using the additional packages Hmisc 4.1-1 (Harrell, 2021), psych 1.8.4 (Revelle, 2018), and cutpointr 1.1.1 (Thiele, 2021).

## 3 Results

**Fig. 3:**
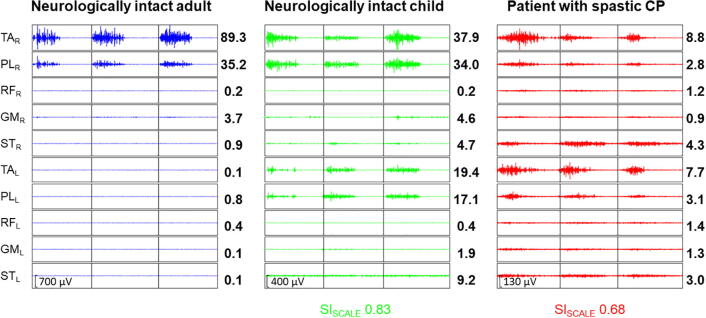
Three examples of EMG recordings during the right ankle dorsiflexion/ plantarflexion test of the SCALE. The numbers represent the root mean square for each muscle that constitute the vector for the calculation of the similarity index. Please note that the axes had to be scaled to display the signal. Abbreviations: TA: m. tibialis anterior, PL: m. peroneus longus, RF: m. rectus femoris, GM: m. gastrocnemius medialis, ST: m. semitendinosus; r: right; l: left; SI_SCALE_: Similarity Index, recorded during the SCALE: Selective Control Assessment of the Lower Extremity.

Fifty-six children, i.e., 24 with spastic and mixed types of CP and 31 neurologically intact children, gave informed consent to participate in this study. Characteristics describing the participants are presented in Table 1. Regarding the MAS, 22.4% (43/192) of the tested movements were scored 1 and 10.9% (21/192) equal or higher than 2. In addition, a descriptive summary of the reference group (31 neurological intact adults; 15 females; median age of 33.9 years [1st; 3rd quartile: 27.5; 38.4]), on which the calculation of the SI was based, is included in Appendix A. Fig. 3 shows examples of EMG recordings during right ankle movements of the SCALE and the extracted values.

**Table 1:** Participants’ characteristics.

	Children with CP	Neurologically intact children
N	24	31
Age (years)	10.56 [8.68,15.28]	10.98 [8.68,13.72]
Sex (male/female)	14/10	15/16
GMFCS level (I/II/III/IV)	9/4/5/6	NT
Leg involvement (bilateral/unilateral)	20/4	NT

The table displays the median [1st; 3rd quartile]. Abbreviations: CP: cerebral palsy; GMFCS: Gross Motor Function Classification System; NT: Not tested.

### 3.1 Concurrent validity

**Fig. 4:**
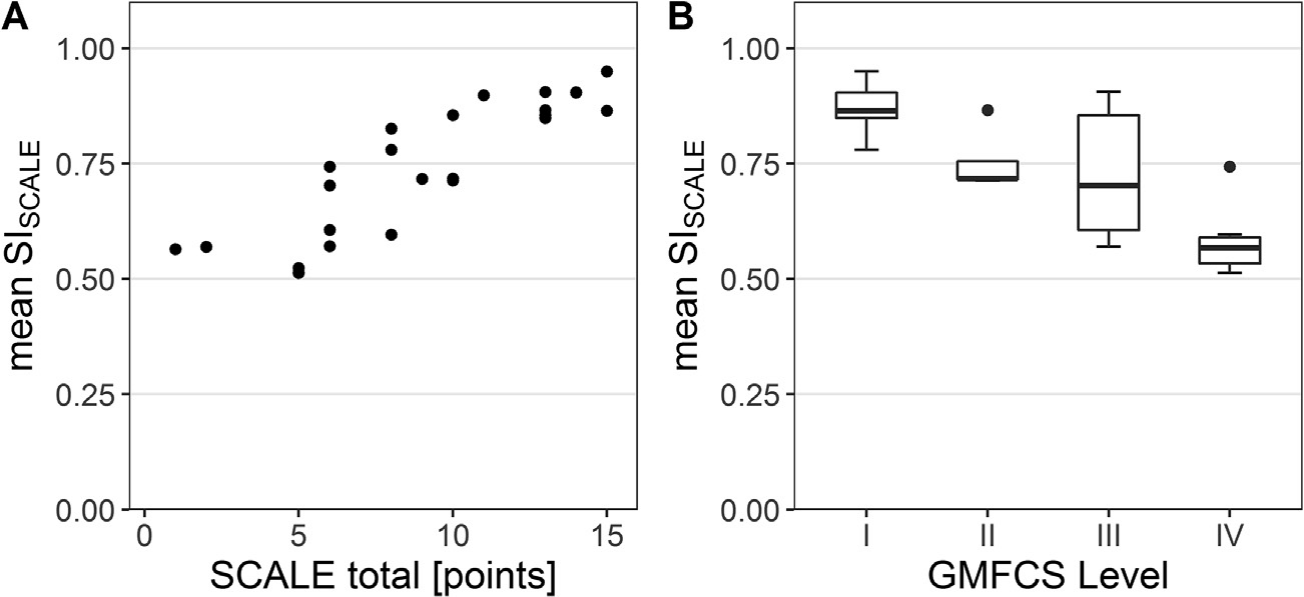
Concurrent validity of total scores. A: Scatterplot of the SCALE and SI_SCALE_. B: Boxplots of the SI_SCALE_ for each GMFCS level. Abbreviations: GMFCS: Gross Motor Function Classification System; SI_SCALE_: Similarity Index, recorded during the SCALE: Selective Control Assessment of the Lower Extremity.

The Spearman’s rank correlations for the total scores (ρ = 0.90, p < .001, Fig. 4) and leg scores (ρ > 0.75, p < .001) were strong. For individual joints, Kendalĺs rank correlations between the SI_SCALE_ and the SCALE were significant and moderate (ρ ranged from 0.37 to 0.57), except for two weak and non-significant correlation coefficients for the less affected ankle and the more affected knee joint (Table 2). There was a strong (negative) correlation between the total SI_SCALE_ and the GMFCS level (ρ = −0.74, p < .001, Fig. 4).

**Table 2:** Convergent and discriminative validity SI_SCALE._

		Children with CP		Correlation		Neurologically intact children	Discrimination of groups
		SI_SCALE_	SCALE	τ/ρ	p value	SI_SCALE_	p value*
**less affected/dominant**	**hip**	0.77 [0.65,0.93]	1.0 [1.0,2.0]	τ = 0.40	0.018	0.98 [0.89,0.98]	<0.001
	**knee**	0.79 [0.61,0.93]	1.0 [1.0,2.0]	τ = 0.49	0.004	0.95 [0.86,0.96]	0.001
	**ankle**	0.91 [0.68,0.98]	1.5 [1.0,2.0]	τ = 0.26	0.116	0.97 [0.94,0.99]	0.016
	**STJ**	0.85 [0.63,0.94]	1.0 [0.0,2.0]	τ = 0.57	<0.001	0.95 [0.91,0.97]	<0.001
	**leg**	0.85 [0.67,0.90]	5.5 [3.75,7.0]	ρ = 0.76	<0.001	0.94 [0.89,0.96]	<0.001

**more affected/non-dominant**	**hip**	0.79 [0.64,0.85]	1.0 [1.0,2.0]	τ = 0.37	0.031	0.97 [0.90,0.98]	<0.001
	**knee**	0.73 [0.55,0.92]	1.0 [1.0,2.0]	τ = 0.29	0.085	0.95 [0.86,0.96]	0.005
	**ankle**	0.85 [0.54,0.94]	1.0 [0.0,2.0]	τ = 0.41	0.014	0.97 [0.88,0.99]	<0.001
	**STJ**	0.68 [0.54,0.91]	1 [0.0,1.0]	τ = 0.50	0.003	0.94 [0.92,0.97]	<0.001
	**leg**	0.76 [0.58,0.85]	4.0 [2.0,6.0]	ρ = 0.80	<0.001	0.93 [0.90,0.96]	<0.001

**total**		0.76 [0.60,0.86]	9.5 [6.0,13.0]	ρ = 0.90	<0.001	0.93 [0.91,0.96]	<0.001

Descriptive values of the SI_SCALE_ and SCALE represent medians [1st; 3rd quartiles]. Abbreviations: CP: cerebral palsy; SI_SCALE_: Similarity Index, recorded during the SCALE: Selective Control Assessment of the Lower Extremity; ρ: Spearman’s rho; STJ: Subtalar joint. *Please note, to account for multiple comparisons, p-values below 0.004 were considered statistically significant.

### 3.2 Discriminative validity

Concerning discriminative validity, SI_SCALE_ scores (separate joints, legs, and total) were significantly lower in children with CP compared to their neurologically intact peers, except for the less affected/dominant ankle and the more affected/non-dominant knee (Table 2). Due to the multiple (11) comparisons, a corrected alpha level of 0.004 was applied.

A majority of the children showed lower SI_SCALE_ scores on the more affected side. However, the difference between the more and less affected leg did not reach significance in either group of children with bilateral or unilateral CP.

The area under the ROC curve for discriminating normal from impaired SVMC was 0.80 (95% confidence interval 0.73–0.85). A SI_SCALE_ of 0.76 could best separate these groups with a sensitivity of 62% and specificity of 85%.

### 3.3 Reliability

Please note that only 20 data sets could be analyzed for reliability testing, as four patients did not participate in the second measurement due to illness or scheduling conflicts. With ICC values exceeding 0.9 for the total SI_SCALE_ score, 0.8 for each leg, and 0.7 for most joint SI_SCALE_ scores, the test–retest reliability was in a moderate to good range. The MDC was lowest for the total and leg scores (≤0.17) and varied between 0.25 and 0.38 for the individual joints (Table 3). For the total score, an MDC of 0.09 corresponds to 13.1% of the grand mean and all of the 20 children could improve by the MDC without reaching the maximum score 1 of the SI_SCALE_.

**Table 3:** Test-retest reliability SI_SCALE._

		Descriptives			Relative reliability		Absolute reliability	
n = 20		mean 1 (SD)	mean 2 (SD)	p value*	ICC (2.1)	95% CI	SEM	MDC_95_
**less affected**	**hip**	0.73 (0.19)	0.68 (0.22)	0.095	0.75	[0.48,0.89]	0.10	0.28
	**knee**	0.73 (0.19)	0.78 (0.16)	0.191	0.66	[0.33,0.85]	0.10	0.29
	**ankle**	0.80 (0.20)	0.82 (0.19)	0.344	0.71	[0.41,0.88]	0.11	0.29
	**STJ**	0.78 (0.17)	0.78 (0.18)	0.675	0.70	[0.38,0.87]	0.10	0.27
	**leg**	0.76 (0.15)	0.76 (0.15)	0.365	0.84	[0.64,0.93]	0.06	0.17

**more affected**	**hip**	0.68 (0.18)	0.68 (0.17)	0.387	0.78	[0.52,0.90]	0.08	0.23
	**knee**	0.69 (0.19)	0.69 (0.19)	0.732	0.82	[0.60,0.92]	0.08	0.23
	**ankle**	0.69 (0.27)	0.67 (0.28)	0.121	0.76	[0.49,0.90]	0.14	0.38
	**STJ**	0.64 (0.24)	0.64 (0.23)	0.825	0.86	[0.69,0.94]	0.09	0.25
	**leg**	0.68 (0.15)	0.67 (0.18)	0.251	0.88	[0.73,0.95]	0.06	0.16

**total**		0.72 (0.13)	0.72 (0.14)	0.560	0.94	[0.85,0.97]	0.03	0.09

Abbreviations: SI_SCALE_: Similarity Index, recorded during the Selective Control Assessment of the Lower Extremity; ICC, Intra-class Correlation Coefficient; CI, Confidence Interval; SD, Standard Deviation; SEM, Standard Error of Measurement; MDC_95_, Minimum Detectable Change at 95% confidence level; STJ: Subtalar joint; *p value of Wilcoxon signed rank test for systematic error between test and retest SI_SCALE_. Please note, we could include data from only 20 participants in the reliability analysis.

## 4 Discussion

The aim of this clinimetric study was to investigate the validity and reliability of the SI_SCALE._ To the authors’ knowledge, this was the first study that applied the SI approach to patients with a neurological diagnosis other than spinal cord injury (Lee et al., 2004, Lim et al., 2005, Lim and Sherwood, 2005).

Generally, we could confirm the hypotheses we had formulated to investigate the concurrent validity. In line with our hypotheses, the correlations between the SI_SCALE_ and the SCALE for the summed scores (leg and total score) were strong, indicating that these measures quantify a similar construct. However, the correlations were lower than expected for the individual joints. We assume that this finding has a statistical cause and has to do with the limited ordinal range of grading each single joint (i.e., a SCALE score of 0, 1, or 2) compared to the summed scores. Differences between the SCALE and the SI_SCALE_ can also arise from the fact that the SI_SCALE_ is based on sEMG measurements whereas the SCALE reflects the observation of joint movements. Therefore, the SI can capture signs of reduced SVMC not resulting in visible movement, typically co-contraction.

The correlation of the SI_SCALE_ total score with the GMFCS level is consistent with previous results of the original SCALE and also confirms our concurrent validity hypothesis (Fowler et al., 2009, Balzer et al., 2016). While the correlation indicates that children with higher SVMC have better mobility, a lower correlation with the GMFCS than with the SCALE was anticipated because the GMFCS is not a specific measure of SVMC.

Only the first of the hypotheses formulated for the discriminative validity, i.e., that SI_SCALE_ scores of children with CP will be different from those of neurologically intact children, could be accepted. It confirms previous observations that children with CP experience impairments in SVMC (Koerte et al., 2009).

Concerning our second discriminative validity hypothesis, we found no significant differences between the more and less affected leg. We explain this with the convenience sampling of our participants resulting in only four children with unilateral brain injuries in this study. In our center, particularly children with major motor impairments are admitted for rehabilitation, for example, to undergo robot-assisted treadmill training. This results in an overrepresentation of children with bilateral spastic CP (Van Hedel et al., 2018), and the small number of participants with unilateral CP significantly affected the statistical power for such a discriminative analysis.

In contrast to previous results indicating proximal–distal concordance (based on SCALE assessments) (Fowler et al., 2010, Balzer et al., 2016), the SI_SCALE_ scores did not differ between adjacent joint pairs. This discrepancy could be caused by the slightly different constructs measured by the SI_SCALE_ and SCALE, i.e., muscle activation versus joint movement, respectively. The SI_SCALE_ can measure SVMC independently from observable joint movement, which allows for a more precise assessment of SVMC, particularly for joints with a small range of motion. For such joints, i.e., merely lower limb distal joints, it is difficult to observe whether the range of motion is above or below 50% of the active range of motion (which is one grading criterion of the SCALE). Moreover, the small differences previously found between single joint SCALE scores (mainly between hip, knee, and ankle joint and less between ankle, STJ, and toes) only exist on group level. For an individual, they cannot be observed since the SCALE only grades absent, impaired, or normal SVMC, indicating that the clinical relevance of the distal-proximal concordance might be overestimated. This difference in the measured constructs can also explain a good but not excellent AUC value.

An ICC value of 0.94 indicates that the total score of the SI_SCALE_ is highly reproducible, and all children could improve by the MDC without surpassing the maximum score. Nevertheless, future studies are needed to establish whether an MDC of 0.09 (total SI_SCALE_) is clinically acceptable. On the one hand, we need to determine whether therapies that aim to improve SVMC achieve such an improvement or more. On the other hand, patients' and rehabilitation specialists’ perceptions about clinically important differences need to be investigated to establish clinically meaningful differences, which should exceed the MDC.

### 4.1 Methodological considerations

One consideration is that spasticity might have occurred during testing, for example, in the antagonistic muscle, which would have affected the SI_SCALE_ score. While we cannot exclude that spasticity-induced EMG activity might have been recorded during the measurements, we think the risk is relatively low because we performed the movements slowly and only few patients had MAS scores above 1.

Second, we changed the original SCALE testing position of the hip from side-lying into sitting. While this improved the quality of the sEMG signals, the participant had to move the leg against gravity and thus required a higher amount of muscle strength. Such an increased effort might have caused more involuntary muscle activity (Addamo et al., 2007). Yet, as can be seen in Table 2, Table 3, the changed position did not result in poorer SI_SCALE_ scores nor reliability results for the hip joint compared to the other joints. Another limitation was the number of participants. Although we had aimed for a sample size of at least 30 participants, data of only 24 participants could be collected within the recruitment period due to the in- and exclusion criteria. Therefore, we consider this study a preliminary evaluation. Lastly, the sEMG signal is influenced by anatomical and morphological factors (e.g., muscle size, tissue between the electrode and the muscle). Despite that we standardized the positioning of the electrodes as much as possible (i.e., in line with the SENIAM guidelines), individual differences of these properties, i.e., between patients or between patients and healthy children, could have affected the recordings and thereby the analyses and comparisons.

### 4.2 Clinical implications

As discussed above, the SI_SCALE_ quantifies SVMC via muscle activation independent of joint movement and muscle strength. This allows measuring SVMC even in participants with considerable muscle weakness. While the SCALE rates the absence of joint movement as ‘unable SVMC (0)’, the SI_SCALE_ can distinguish between participants with no movement but physiological muscle activation patterns and those with no movement and less physiological muscle activation. This increased sensitivity might serve to predict and optimize physiotherapeutic treatment regimes. For example, if movement is almost absent due to reduced strength, but the SI_SCALE_ indicates the preservation of SVMC, strength training might be advisable. In contrast, in children with low SI_SCALE_ and poor strength, both aspects are relevant. The therapy should focus on increasing muscle strength combined with or followed by a training of selective movement patterns. For this case, we still have to find possibilities to train SVMC specifically (Fahr et al., 2020).

The SI_SCALE_ score does not indicate why SVMC is reduced. By analyzing the individuaĺs response vector, one can distinguish between impaired SVMC due to co-activation of muscles in the same extremity, mirror activity on the contralateral side, or a combination of both. This is essential for identifying the exact impairments underlying a reduced SVMC score and proposing treatment contents. Moreover, a patient’s SI_SCALE_ should be interpreted relative to the score of an age-matched group of neurologically intact children. A tentative example of how the output of the SI_SCALE_ could be clinically used is shown exemplarily in Appendix B. Currently, the reference data should be considered with caution as they are based on reference values of 31 neurological intact adults and 31 typically developing children only. In the near future, such output would allow clinicians to detect the underlying impairments in SVMC in more detail and deploy a more refined therapy program.

Concerning the clinical utility of SI_SCALE_, its regular application might be feasible in medical centers where sEMG and qualified personnel are available. Its application and analysis using a customized data analysis program will take around 30 min.

## 5 Conclusion

The SI_SCALE_ measures SVMC based on sEMG signals independently from joint movement. This study showed encouraging preliminary findings regarding the validity and reliability of the SI_SCALE_. To apply the SI_SCALE_ as an outcome measure for detecting therapy-induced changes, evaluations within a larger sample and responsiveness-testing are needed.

## Appendix A Supplementary data

Supplementary data 1 Participants’ characteristics of the adult reference group.

Supplementary data 2 Detailed output of the SI_SCALE_.
