# Supplementary material for: Validity and reliability of an electromyography-based similarity index to quantify lower extremity selective voluntary motor control in children with cerebral palsy
Source: Clin Neurophysiol Pract. 2022 Mar 17;7:107–14. doi: 10.1016/j.cnp.2022.03.003 (PMC8967969; doi:10.1016/j.cnp.2022.03.003)
Supplement: Supplementary data 1 — Participants’ characteristics of the adult reference group. [file mmc1.docx]

**Supplementary Table S1.** Participants’ characteristics of the adult reference group for the calculation of the similarity index.

|  |  | **Neurologically intact adults** |
| --- | --- | --- |
| **N** |  | 31 |
| **Age (years)** |  | 33.94 [27.53,38.43] |
| **Sex (male/female)** |  | 16 / 15 |
| **SI_SCALE_** | **dominant** | 0.99 [0.92,0.97] |
|  | **non-dominant** | 0.95 [0.92,0.97] |
|  | **total** | 0.95 [0.92,0.96] |

*The table displays the median [1^st^; 3^rd^ quartile]. Abbreviations: SI_SCALE_: Similarity Index (recorded during) Selective Control Assessment of the Lower Extremity.*
