# Supplementary material for: Validity and reliability of an electromyography-based similarity index to quantify lower extremity selective voluntary motor control in children with cerebral palsy
Source: Clin Neurophysiol Pract. 2022 Mar 17;7:107–14. doi: 10.1016/j.cnp.2022.03.003 (PMC8967969; doi:10.1016/j.cnp.2022.03.003)
Supplement: Supplementary data 2 — Detailed output of the SISCALE. [file mmc2.pdf]

Detailed output of the SI_{SCALE}

Overview of the SI_{SCALE}

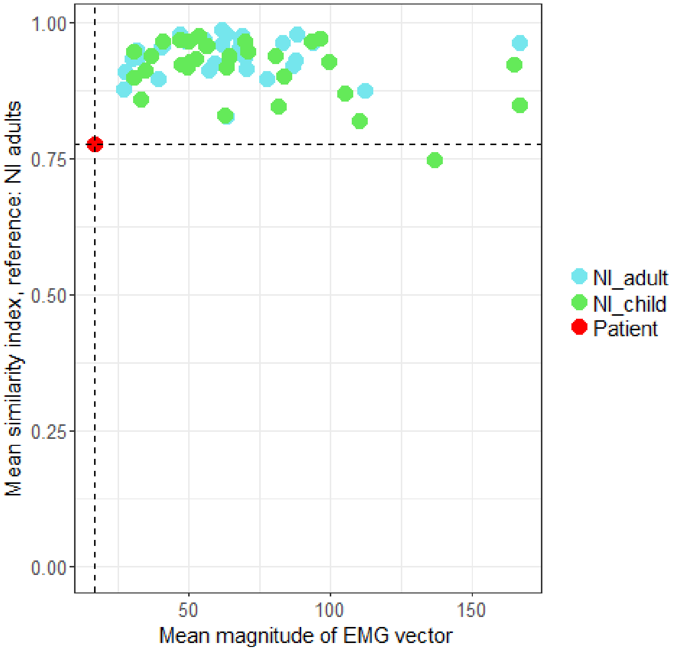

		NIA	NIC	Patient	Percentile [%]
n		31	5	1	
Mean age		33.73	16.32	16.12	
**Mean of all SI_{SCALE}**		0.94	0.92	0.78	
**SI of individual joints**					
Hip joint	*less affected side*	0.93	0.93	0.74	8.36
Knee joint		0.93	0.92	0.95	66.58
Ankle joint		0.96	0.91	0.59	0.8
Subtalar joint		0.94	0.91	0.94	66.72
Hip joint	*more affected side*	0.94	0.95	0.94	37.04
Knee joint		0.95	0.93	0.86	20.91
Ankle joint		0.95	0.88	0.54	8.98
Subtalar joint		0.91	0.95	0.64	0.42

Abbreviations: EMG: Electromyography, NIA: Neurologically intact adults, NIC: Neurologically intact children, SI_{SCALE}: Similarity index, recorded during the SCALE: Selective control assessment of the lower extremity.

Contribution of the muscles to the SI_{SCALE} vectors

Hip joint, *less affected side*

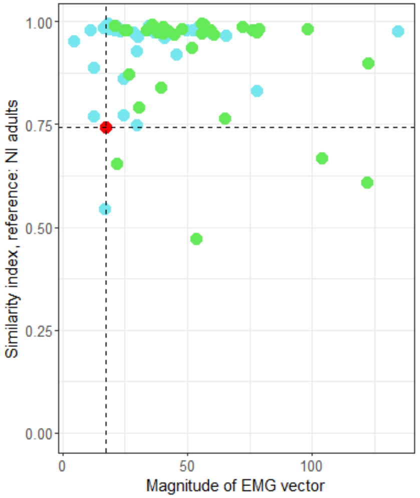

	NIA	NIC	Patient
**SI_{SCALE}**	0.93	0.93	0.74
m. tibialis anterior *ipsi.*	0.08	0.03	0.01
m. peroneus *ipsi.*	0.05	0.02	0.01
m. rectus femoris *ipsi.*	0.88	0.89	0.6
m. gastrocnemius med. *ipsi.*	0.06	0.1	0.01
m. semitendinosus *ipsi.*	0.16	0.09	0.26
m. tibialis anterior *contra.*	0.03	0	0.21
m. peroneus *contra.*	0.01	0	0.26
m. rectus femoris *contra.*	0.04	0.02	0.36
m. gastrocnemius med. *contra.*	0.03	0.04	0.06
m. semitendinosus *contra.*	0.24	0.29	0.57
SCALE score		2	1

Knee joint, *less affected side*

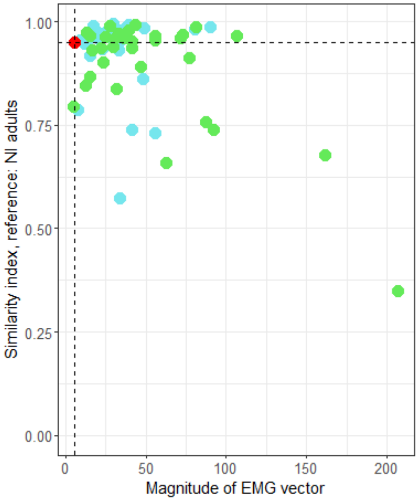

	NIA	NIC	Patient
**SI_{SCALE}**	0.93	0.92	0.95
m. tibialis anterior *ipsi.*	0.25	0.08	0.02
m. peroneus *ipsi.*	0.08	0.05	0.13
m. rectus femoris *ipsi.*	0.88	0.92	0.99
m. gastrocnemius med. *ipsi.*	0.1	0.06	0.05
m. semitendinosus *ipsi.*	0.08	0.1	0.05
m. tibialis anterior *contra.*	0.03	0.01	0.01
m. peroneus *contra.*	0.01	0	0.01
m. rectus femoris *contra.*	0.03	0.01	0.05
m. gastrocnemius med. *contra.*	0.02	0.02	0.01
m. semitendinosus *contra.*	0.06	0.21	0.05
SCALE score		2	1

Ankle joint, *less affected side*

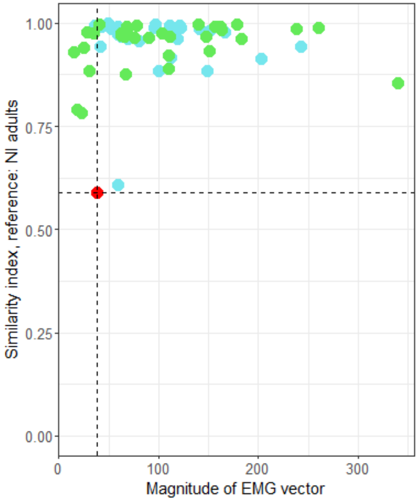

	NIA	NIC	Patient
**SI_{SCALE}**	0.96	0.91	0.59
m. tibialis anterior *ipsi.*	0.87	0.69	0.25
m. peroneus *ipsi.*	0.39	0.66	0.96
m. rectus femoris *ipsi.*	0.02	0.05	0.05
m. gastrocnemius med. *ipsi.*	0.13	0.08	0.05
m. semitendinosus *ipsi.*	0.01	0.01	0.01
m. tibialis anterior *contra.*	0.01	0.01	0.02
m. peroneus *contra.*	0	0.01	0.01
m. rectus femoris *contra.*	0	0.01	0.01
m. gastrocnemius med. *contra.*	0.01	0.09	0
m. semitendinosus *contra.*	0.02	0.01	0
SCALE score		1.8	2

Subtalar joint, *less affected side*

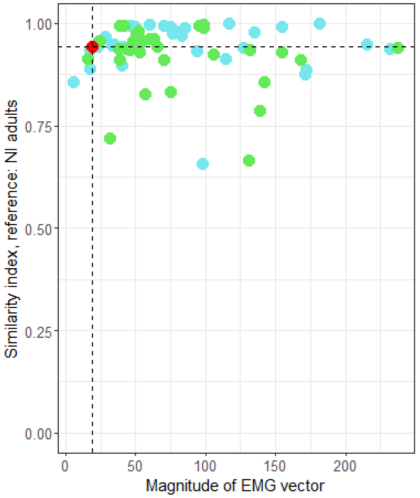

	NIA	NIC	Patient
**SI_{SCALE}**	0.94	0.91	0.94
m. tibialis anterior *ipsi.*	0.47	0.26	0.19
m. peroneus *ipsi.*	0.82	0.9	0.96
m. rectus femoris *ipsi.*	0.02	0.04	0.06
m. gastrocnemius med. *ipsi.*	0.1	0.19	0.18
m. semitendinosus *ipsi.*	0.02	0.03	0.01
m. tibialis anterior *contra.*	0.01	0	0.04
m. peroneus *contra.*	0.01	0	0.01
m. rectus femoris *contra.*	0	0	0.02
m. gastrocnemius med. *contra.*	0.02	0.01	0
m. semitendinosus *contra.*	0.02	0.04	0
SCALE score		2	2

Abbreviations: cont: Contralateral, EMG: Electromyography, ipsi: Ipsilateral, NIA: Neurologically intact adults, NIC: Neurologically intact children, SI_{SCALE}: Similarity index, recorded during the SCALE: Selective control assessment of the lower extremity.

Hip joint, *more affected side*

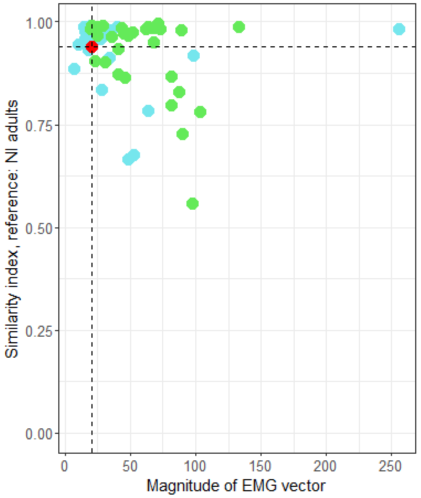

	NIA	NIC	Patient
**SI$_{SCALE}$**	0.94	0.95	0.94
m. tibialis anterior *ipsi.*	0.11	0.03	0.42
m. peroneus *ipsi.*	0.04	0.02	0.04
m. rectus femoris *ipsi.*	0.89	0.89	0.88
m. gastrocnemius med. *ipsi.*	0.09	0.08	0.01
m. semitendinosus *ipsi.*	0.17	0.16	0.12
m. tibialis anterior *contra.*	0.03	0.01	0.02
m. peroneus *contra.*	0.02	0.01	0.01
m. rectus femoris *contra.*	0.04	0.01	0.01
m. gastrocnemius med. *contra.*	0.05	0.02	0.01
m. semitendinosus *contra.*	0.19	0.3	0.18
SCALE score		2	1

Knee joint, *more affected side*

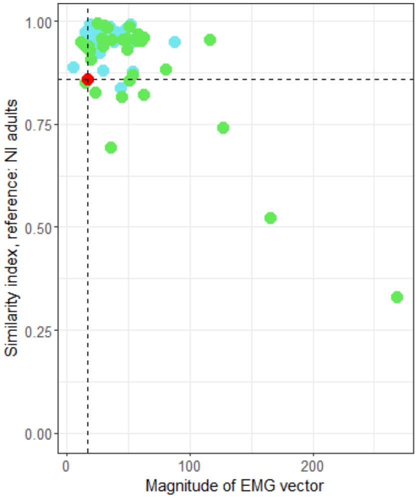

	NIA	NIC	Patient
**SI$_{SCALE}$**	0.95	0.93	0.86
m. tibialis anterior *ipsi.*	0.25	0.09	0.05
m. peroneus *ipsi.*	0.09	0.02	0.55
m. rectus femoris *ipsi.*	0.91	0.94	0.82
m. gastrocnemius med. *ipsi.*	0.07	0.08	0.17
m. semitendinosus *ipsi.*	0.09	0.11	0.02
m. tibialis anterior *contra.*	0.02	0.01	0.01
m. peroneus *contra.*	0.01	0.01	0
m. rectus femoris *contra.*	0.02	0.01	0
m. gastrocnemius med. *contra.*	0.04	0.04	0
m. semitendinosus *contra.*	0.06	0.15	0
SCALE score		2	1

Ankle joint, *more affected side*

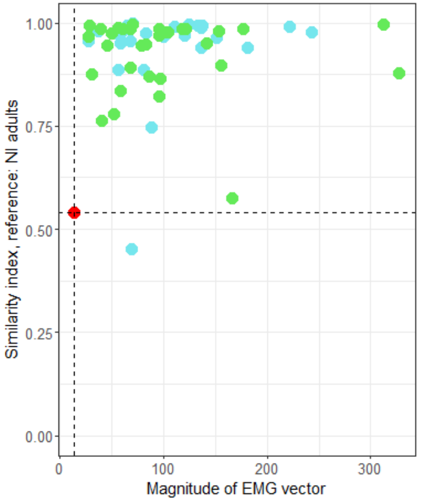

	NIA	NIC	Patient
**SI$_{SCALE}$**	0.95	0.88	0.54
m. tibialis anterior *ipsi.*	0.87	0.75	0.14
m. peroneus *ipsi.*	0.34	0.4	0.82
m. rectus femoris *ipsi.*	0.04	0.05	0.43
m. gastrocnemius med. *ipsi.*	0.14	0.26	0.28
m. semitendinosus *ipsi.*	0.01	0.01	0.18
m. tibialis anterior *contra.*	0.02	0	0.01
m. peroneus *contra.*	0.01	0.01	0
m. rectus femoris *contra.*	0	0	0
m. gastrocnemius med. *contra.*	0.03	0.02	0.01
m. semitendinosus *contra.*	0.02	0	0.05
SCALE score		1.8	0

Subtalar joint, *more affected side*

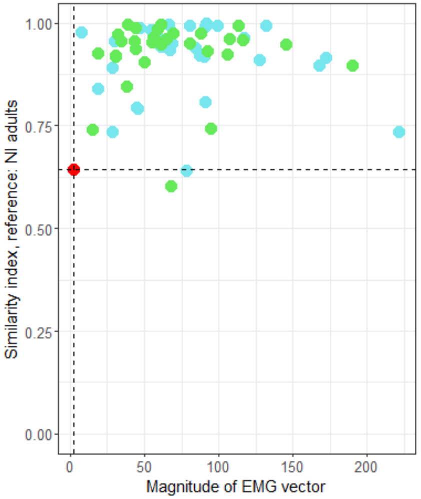

	NIA	NIC	Patient
**SI$_{SCALE}$**	0.91	0.95	0.64
m. tibialis anterior *ipsi.*	0.51	0.34	0.15
m. peroneus *ipsi.*	0.75	0.87	0.6
m. rectus femoris *ipsi.*	0.03	0.06	0.14
m. gastrocnemius med. *ipsi.*	0.12	0.26	0.44
m. semitendinosus *ipsi.*	0.02	0.03	0.63
m. tibialis anterior *contra.*	0.01	0.01	0.04
m. peroneus *contra.*	0.02	0.04	0.02
m. rectus femoris *contra.*	0.01	0.01	0.03
m. gastrocnemius med. *contra.*	0.05	0.02	0.02
m. semitendinosus *contra.*	0.02	0	0.05
SCALE score		2	0
